# Altered Individualized Structural Covariance Networks Underlie Depressive and Anxiety Symptoms in Chronic Insomnia Disorder: A Dynamic Time Warping Graph Theory Study

**DOI:** 10.1002/brb3.71830

**Published:** 2026-10-16

**Authors:** Liang Gong, Duan Liu, Bei Zhang, Haoyu Li, Yuan He, Ronghua Xu, Wenjun Zhou, Chunhua Xi

**Affiliations:** ^1^ Department of Neurology West China School of Medicine Sichuan University Sichuan University affiliated Chengdu Second People's Hospital Chengdu China; ^2^ School of Computer Science and Software Engineering Southwest Petroleum University Chengdu China; ^3^ Department of Neurology The Third Affiliated Hospital of Anhui Medical University The First People's Hospital of Hefei Hefei China

**Keywords:** anxiety, depression, graph theory, individualized structural covariance network, Insomnia

## Abstract

**Purpose:**

Chronic insomnia disorder (CID) is highly comorbid with depression and anxiety, yet the neurostructural basis of this affective comorbidity remains unclear. This study aimed to investigate individualized structural covariance network (iSCN) alterations in CID and their associations with depressive and anxiety symptoms using a novel dynamic time warping (DTW) approach.

**Methods:**

A total of 257 CID patients and 247 good sleep controls (GSCs) underwent T1‐weighted structural magnetic resonance imaging. Individualized SCNs were constructed based on gray matter volume using the DTW algorithm to quantify interregional morphological similarity. Graph theory analysis was applied to assess global and nodal network topological properties. Clinical symptoms were evaluated using standardized scales (PSQI, SDS, SAS).​

**Findings:**

Compared with GSCs, patients with CID exhibited significant disruptions in global network topology, including reduced local efficiency, global efficiency, and small‐world properties (Gamma and Sigma). At the nodal level, both increased and decreased nodal degree and betweenness centrality were observed in regions involved in emotion regulation, arousal, and sensory processing, including the orbitofrontal cortex, amygdala, thalamus, and caudate nucleus. These nodal metrics were significantly associated with depressive and anxiety symptom severity after correction for multiple comparisons.

**Conclusion:**

Our findings demonstrate that CID is characterized by disrupted iSCN organization, and that nodal alterations within emotion‐, arousal‐, and sensory‐related regions are associated with the severity of depressive and anxiety symptoms. These findings identify altered large‐scale structural coordination as a neural correlate of affective symptom burden in CID.

## 1 Introduction

Chronic insomnia disorder (CID) is a highly prevalent sleep disorder characterized by persistent difficulties in sleep initiation, maintenance, or early morning awakening, accompanied by significant daytime impairment and distress (Buysse 2013). Beyond its core sleep‐related symptoms, CID exhibits high comorbidity with affective disorders, particularly depression and anxiety (Gong et al. 2021; Morin et al. 2015; Peng et al. 2024). This common co‐occurrence suggests potential shared neurobiological mechanisms, yet the precise neural substrates linking disrupted sleep with emotional dysregulation remain inadequately elucidated.

Traditional neuroimaging investigations of insomnia have primarily used voxel‐ or surface‐based morphometry and resting‐state functional connectivity (rs‐FC) to identify alterations in brain structure and function. Functional imaging studies have implicated distributed networks related to hyperarousal, including the default mode network (DMN), salience network (SN), sensorimotor network (SMN), dorsal attention network (DAN), and ascending arousal network (AAN) (Chen et al. 2025; Cheng et al. 2022; Fasiello et al. 2022; Gong, He, et al. 2023; Schiel et al. 2020; Yu et al. 2018). Structural studies have reported lower gray matter volume in the hippocampus, orbitofrontal cortex, and anterior cingulate cortex (Altena et al. 2010; Falgàs et al. 2021), whereas gray matter hypertrophy in the orbitofrontal cortex and amygdala has also been described (Gong et al. 2019; Yu et al. 2020). However, most studies focus on group‐level effects and may overlook substantial individual variability in neuroanatomy and clinical presentation. Moreover, a recent large‐scale coordinated meta‐analysis across three cohorts did not identify significant associations between insomnia symptoms and cortical or subcortical volumes (Weihs et al. 2023). Thus, how the structural organization of brain networks relates to specific affective symptoms in CID remains incompletely understood.

The structural covariance network (SCN) provides a framework for examining coordinated neurodevelopmental and neuroplastic variation across distributed brain regions by quantifying interregional similarity in morphological features such as gray matter volume (Kuo et al. 2020; Suh et al. 2016). Most conventional SCN studies estimate covariance at the group level and therefore cannot directly characterize individual network organization (Wu et al. 2017). Recent work has adapted dynamic time warping (DTW) to construct individualized SCNs (iSCNs) (Sun et al. 2024). In this approach, voxelwise gray matter volume values within each anatomical region are treated as regional morphological vectors. DTW uses elastic, nonlinear alignment to identify the minimum cumulative distance between two regional vectors, including vectors of different lengths; a smaller DTW distance indicates greater morphological similarity. Unlike Pearson correlation, which requires elementwise correspondence and quantifies linear covariation, DTW estimates similarity after optimal alignment and can therefore generate a subject‐specific interregional similarity measure without relying on group‐level covariance estimation (Meszlényi et al. 2017; Sun et al. 2024). Combined with graph theory, this approach enables individualized quantification of network integration, segregation, and nodal centrality. A critical question is whether such individualized structural covariance alterations are related to the depressive and anxiety symptoms that commonly accompany CID.

In the present study, we used DTW‐based iSCNs and graph theory to characterize individualized structural network topology in a large sample of patients with CID and GSCs. We hypothesized that global and nodal network topology would differ between groups and that altered nodal metrics would be associated with depressive and anxiety symptom severity in CID.

## 2 Methods

### 2.1 Participants

A total of 257 patients with CID and 247 good sleep controls (GSCs) were recruited from the outpatient department of neurology at Chengdu Second People's Hospital (CSPH) between May 2021 and June 2025, consistent with our earlier research (Gong, He, et al. 2023; Gong et al. 2022; Zhong et al. 2025). The study was approved by the Research Ethics Committee of CSPH (Approval No. 2020021 and 2023061), and written informed consent was obtained from all participants. Inclusion criteria for CID participants were as follows: (1) diagnosis of CID according to the International Classification of Sleep Disorders, Third Edition (Sateia 2014); (2) experience of disrupted sleep (difficulty initiating sleep, maintaining sleep, or early morning awakening) for a minimum of three months; (3) Pittsburgh Sleep Quality Index (PSQI) score > 7 (Backhaus et al. 2002; Gong et al. 2025); (4) no use of hypnotic or antidepressant medications within two weeks preceding neuropsychological assessment and MRI scanning; and (5) age between 18 and 60 years. Exclusion criteria included: (1) other neuropsychiatric disorders, including major depressive disorder or generalized anxiety disorder; (2) severe depressive or anxiety symptoms, defined as a standardized Self‐Rating Depression Scale (SDS) score ≥ 70 or Self‐Rating Anxiety Scale (SAS) score ≥ 70; (3) other sleep disorders, including sleep‐related breathing disorders (e.g., sleep apnea syndrome); (4) substance abuse, including excessive caffeine or alcohol consumption; (5) contraindications for MRI; and (6) brain abnormalities detected on conventional MRI. GSC participants were selected using similar criteria but reported no sleep complaints and had PSQI scores below 7.

### 2.2 Assessments

Diagnostic eligibility for CID and exclusion of major depressive disorder, generalized anxiety disorder, and other major neuropsychiatric disorders were determined by a clinical diagnostic interview conducted by two experienced neurologists (DL and LG), with consensus achieved in all cases. Sleep disturbance severity was quantified using the Pittsburgh Sleep Quality Index (PSQI) (Backhaus et al. 2002; Buysse et al. 1989). Depressive and anxiety symptom severity was quantified using the Zung Self‐Rating Depression Scale (SDS) (Zung et al. 1965) and the Zung Self‐Rating Anxiety Scale (SAS) (Zung 1971), respectively. The SDS and SAS were completed once by each participant as self‐rating measures of dimensional symptom burden rather than as diagnostic instruments. Participants with standardized SDS or SAS scores ≥ 70 were excluded as described above.

### 2.3 Image Acquisition and Preprocessing

MRI scanning was conducted for all participants using a 3.0‐Tesla scanner (GE Healthcare Discovery MR750, Milwaukee, WI, USA). High‐resolution T1‐weighted structural images were acquired with a spoiled gradient‐recalled echo sequence using the following parameters: repetition time/echo time = 1900/2.48 ms; flip angle = 7°; acquisition matrix = 256 × 256; field of view = 256 × 256 mm^2^; slice thickness = 1.0 mm (no gap); number of slices = 188.

Preprocessing of structural MRI was performed using the VBM8 toolbox implemented in SPM8 (http://www.fil.ion.ucl.ac.uk/spm) within MATLAB R2012b (The MathWorks Inc., Natick, MA, USA). Each structural image was segmented into gray matter (GM), white matter, and cerebrospinal fluid. Subsequently, DARTEL normalization was applied to transform GM images into Montreal Neurological Institute (MNI) space. Finally, normalized GM images were smoothed with an 8 mm full‐width at half‐maximum Gaussian kernel to improve signal‐to‐noise ratio (Ashburner and Friston 2000).

### 2.4 Individual Structural Covariance Network and Graph Theory Analysis

Network nodes were defined according to the automated anatomical labeling (AAL) atlas (Tzourio‐Mazoyer et al. 2002), comprising 116 regions—90 cortical and subcortical regions and 26 cerebellar regions. Individual structural covariance networks were constructed within each participant using DTW to quantify interregional gray matter morphological similarity (Sun et al. 2024). Briefly, voxelwise gray matter volume values within each AAL region were vectorized. For each pair of regions, DTW constructed a point‐to‐point distance matrix and identified an optimal warping path that minimized cumulative distance between the two regional vectors. Lower DTW distance indicated greater similarity; DTW distances were transformed into similarity values following the procedure described by Sun et al. (2024). This yielded a 116 × 116 individualized association matrix for each participant for subsequent graph‐theory analyses.

Graph theoretical metrics were computed using the GRETNA toolkit (https://www.nitrc.org/projects/gretna). Binary networks were derived by applying sparsity thresholds from 0.05 to 0.40 in steps of 0.05, consistent with prior individual morphological network and DTW‐based iSCN studies (W. Li et al. 2017; Sun et al. 2024). This range samples network topology from sparse to moderately dense configurations while reducing dependence on a single arbitrary threshold. Global network properties included clustering coefficient (Cp), global efficiency (Eg), local efficiency (Eloc), shortest path length (Lp), assortativity, modularity, and small‐world measures. Nodal metrics included nodal degree and betweenness centrality. The area under the curve (AUC) across the full sparsity range was calculated for each network metric and used as the primary threshold‐independent summary measure for subsequent statistical analyses (the flowchart of this study was presented in Figure 1).

**FIGURE 1:**
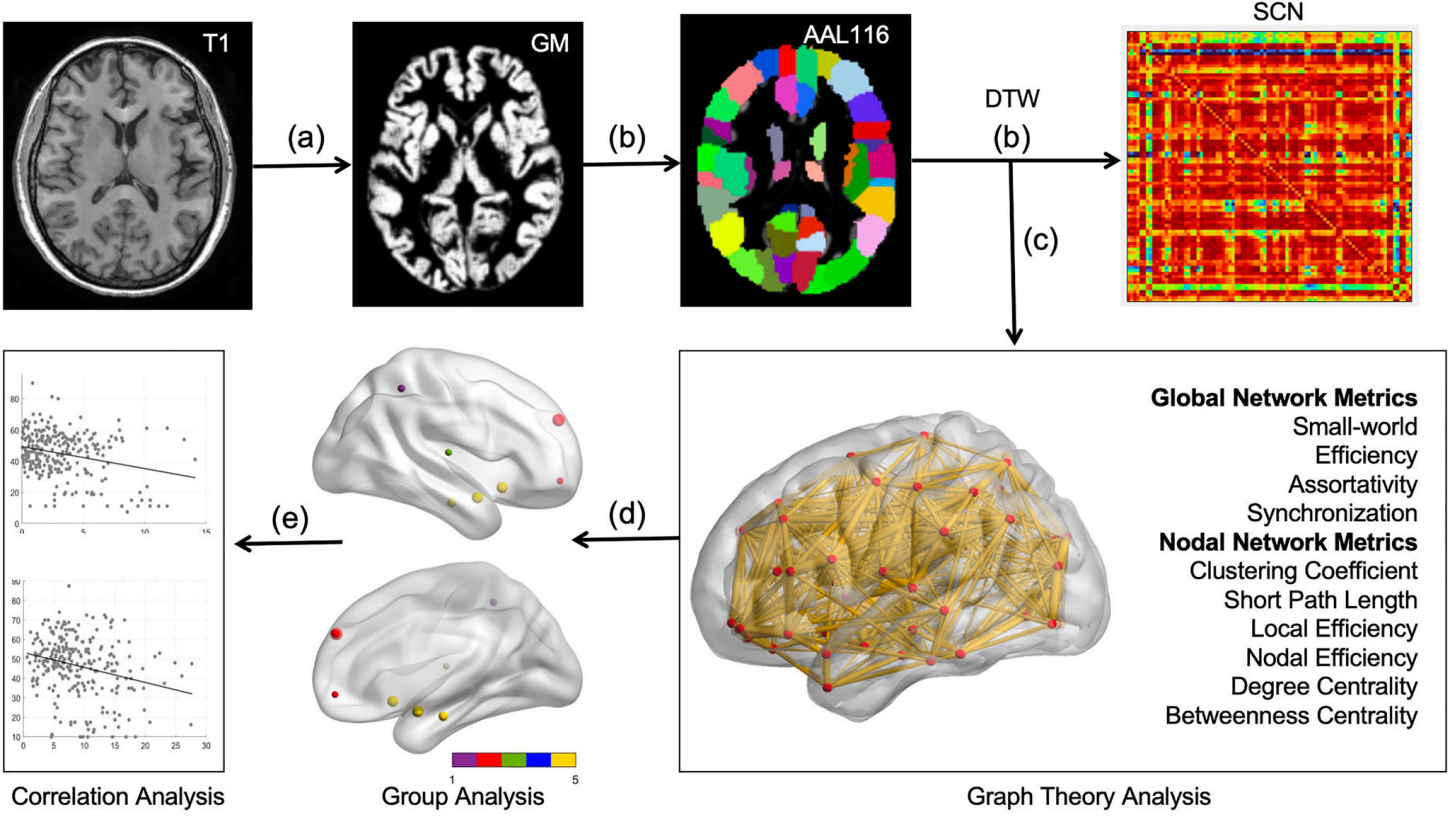
Study workflow. (a) High‐resolution T1‐weighted images were segmented to obtain gray matter maps. (b) Gray matter values were organized according to the AAL parcellation. (c) Pairwise regional morphological similarity was quantified using dynamic time warping (DTW) to construct an individualized structural covariance network (iSCN) for each participant. (d) Graph‐theory metrics were calculated across sparsity thresholds of 0.05–0.40 (step 0.05), and AUC values were compared between 257 patients with chronic insomnia disorder (CID) and 247 good sleep controls (GSCs) using two‐sample t tests with FDR correction. (e) Within the CID group, subject‐level AUC values of significant network metrics were related to PSQI, SDS, and SAS scores using partial correlations adjusted for age, sex, and education, with FDR‐corrected *p* < 0.05 considered significant. AAL, automated anatomical labeling; AUC, area under the curve; FDR, false discovery rate; PSQI, Pittsburgh Sleep Quality Index; SDS, Zung Self‐Rating Depression Scale; SAS, Zung Self‐Rating Anxiety Scale.

### 2.5 Statistical Analysis

Demographic variables and global tissue volumes were compared using two‐sample t tests for continuous variables and a chi‐square test for sex in SPSS 20.0 (IBM Corp., Chicago, IL, USA). Total gray matter volume, total white matter volume, and total intracranial volume (TIV) derived from structural‐image segmentation were included in these between‐group descriptive comparisons. Group differences in global and nodal network AUC metrics between CID and GSC participants were assessed using two‐sample t tests. Cohen's d was used to quantify effect size for between‐group differences. The false discovery rate (FDR) method was applied to correct for multiple comparisons, with two‐sided FDR‐corrected p < 0.05 considered significant.

Partial correlation analyses were conducted within the CID group to examine associations between clinical variables (illness duration, PSQI, SDS, and SAS scores) and the subject‐level AUC values of network metrics that showed significant between‐group differences. Thus, the correlation analyses used each participant's original network metric rather than the magnitude of the group‐level alteration. Age, sex, and years of education were included as covariates. Statistical significance was set at FDR‐corrected *p* < 0.05.

To explore whether structural network metrics statistically mediated the association between insomnia severity and affective symptom burden, we additionally performed exploratory single‐mediator analyses within the CID group. The PSQI score was entered as the independent variable, SDS, or SAS score as the outcome, and each nodal degree or betweenness AUC metric that showed an FDR‐significant association with the corresponding affective score in the primary correlation analysis was entered separately as the mediator. Age, sex, and years of education were included as covariates. Indirect effects (a × b) were estimated using 5,000 bootstrap resamples with 95% confidence intervals, and FDR correction was applied across candidate mediators for each outcome. An indirect effect was considered significant when its bootstrap 95% confidence interval excluded zero and the FDR‐adjusted q value was < 0.05. Because participants with diagnosed major affective disorders and severe SDS/SAS scores were excluded a priori and the remaining symptom burden was analyzed dimensionally, no post hoc threshold‐based affective subgroup analysis was performed.

## 3 Results

### 3.1 Demographic and Clinical Features

Demographic and clinical characteristics are presented in Table 1. The CID and GSC groups did not differ significantly in age or sex (*p* > 0.05). The CID group had more years of education than the GSC group (14.69 ± 3.15 vs. 13.55 ± 3.63 years, *p* < 0.001). As expected, PSQI, SDS, and SAS scores were significantly higher in the CID group (all *p* < 0.001). Mean illness duration in the CID group was 50.41 ± 55.20 months. No significant between‐group differences were observed in total gray matter volume (GSC: 697.04 ± 98.01; CID: 696.42 ± 70.90; *p* = 0.935), total white matter volume (467.63 ± 60.57 vs. 460.89 ± 46.85; *p* = 0.437), or TIV (1476.07 ± 158.73 vs. 1468.58 ± 140.06; *p* = 0.712), indicating no evidence of a gross whole‐brain tissue‐volume difference between groups.

**TABLE 1:** Demographic and clinical characteristics of the study participants.

	GSC(n = 247)	CID(n = 257)	t/χ^2^	Effect Size	*P*
Age	36.78 ± 11.17	35.56 ± 10.94	1.30	d = 0.11​	0.19
Sex (Male/Female)	110/137	102/155	1.21	V = 0.05​	0.16*
Education (years)	13.55 ± 3.63	14.69 ± 3.15	3.75	d = 0.25​	<0.001
PSQI	3.19 ± 1.64	13.06 ± 2.80	41.45	d = 0.63​	<0.001
SAS	37.87 ± 10.78	45.26 ± 14.02	5.30	d = 1.00​	<0.001
SDS	33.35 ± 7.25	46.72 ± 14.77	9.80	d = 0.95​	<0.001
Duration (months)	—	50.41 ± 55.20	—	—	—
Gray matter volume	697.04 ± 98.01	696.42 ± 70.90	0.08​	d = 0.01​	0.935
White matter volume	467.63 ± 60.57	460.89 ± 46.85	0.78​	d = 0.07​	0.437
Total intracranial volume	1476.07 ± 158.73	1468.58 ± 140.06	0.37​	d = 0.03​	0.712

Data are mean ± SD unless otherwise indicated. Continuous variables and global tissue‐volume measures were compared using two‐sample t tests; sex was compared using a chi‐square test (asterisk). Effect sizes are reported as Cohen's d for continuous variables and Cramer's V for sex. GSC, n = 247; CID, n = 257. CID, chronic insomnia disorder; GSC, good sleep controls; PSQI, Pittsburgh Sleep Quality Index; SDS, Zung Self‐Rating Depression Scale; SAS, Zung Self‐Rating Anxiety Scale.

### 3.2 Group Differences in Global Network Topological Properties Between CID and GSC Groups

For global network topology, the CID group showed lower local efficiency (Eloc; t = −3.41, *p* = 0.0007, Cohen's d = 0.30) and global efficiency (Eg; t = −2.56, *p* = 0.011, Cohen's d = 0.23) than the GSC group. The CID group also showed reduced small‐world‐related measures, including Gamma (t = −4.01, *p* = 0.0001, Cohen's d = 0.36), Sigma (t = −4.15, *p* = 3.87 × 10^−^^5^, Cohen's d = 0.37), and Cp (t = −3.50, *p* = 0.0005, Cohen's d = 0.31) (Figure 2).

**FIGURE 2:**
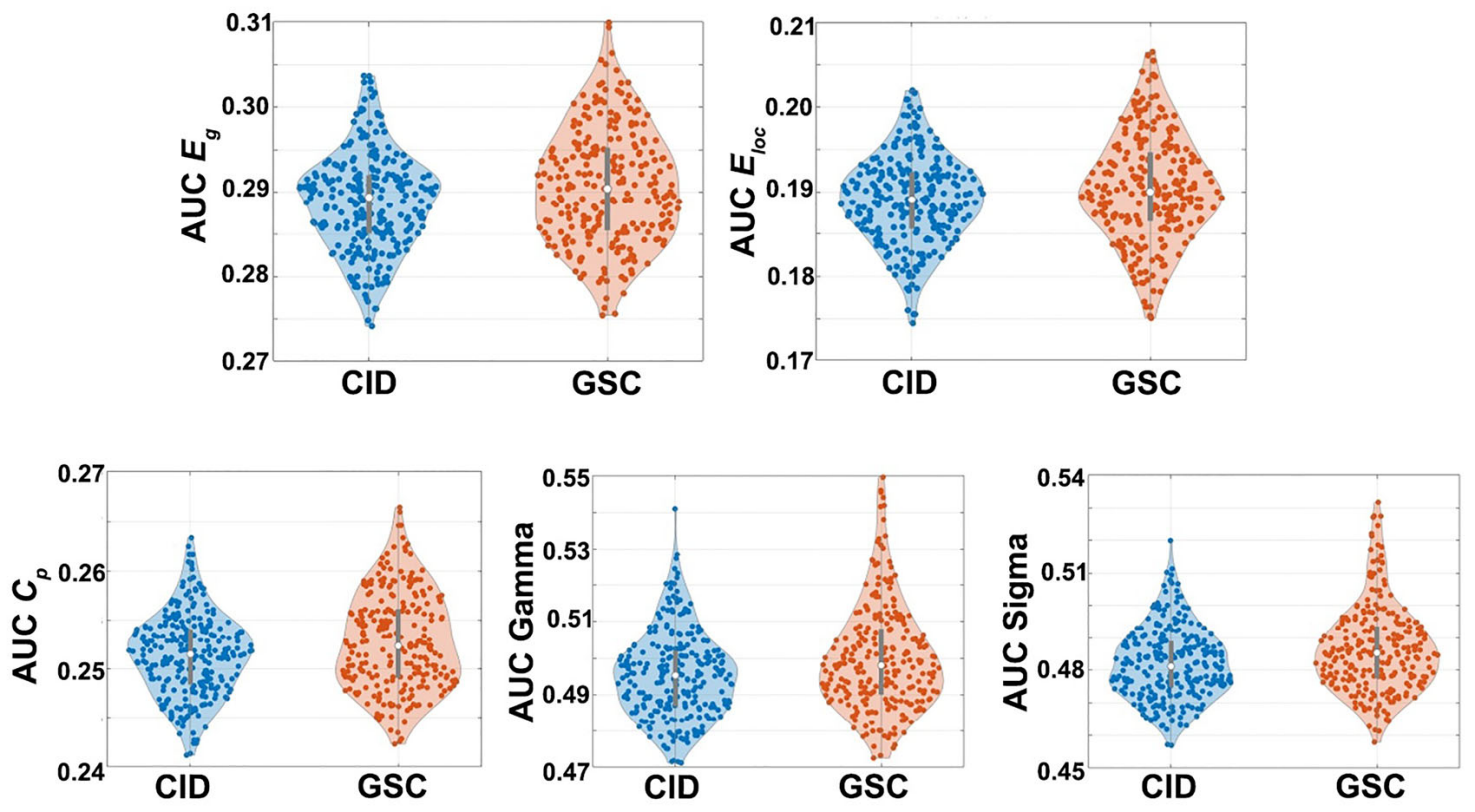
Group differences in global network topological properties. Violin plots show AUC values calculated across sparsity thresholds of 0.05–0.40 (step 0.05) in CID (n = 257) and GSC (n = 247) participants. Two‐sample t tests showed lower Eloc (t = −3.41, *p* = 0.0007, Cohen's d = 0.30), Eg (t = −2.56, *p* = 0.011, Cohen's d = 0.23), Cp (t = −3.50, *p* = 0.0005, Cohen's d = 0.31), Gamma (t = −4.01, *p* = 0.0001, Cohen's d = 0.36), and Sigma (t = −4.15, *p* = 3.87 × 10^−^^5^, Cohen's d = 0.37) in CID. Multiple comparisons were controlled using FDR correction. CID, chronic insomnia disorder; GSC, good sleep controls; AUC, area under the curve; Eg, global efficiency; Eloc, local efficiency; Cp, clustering coefficient.

### 3.3 Group Differences in Nodal Network Topological Properties Between CID and GSC Groups

After FDR correction, the CID group showed both higher and lower nodal degree than the GSC group. Higher nodal degrees was observed in the right inferior parietal lobule (IPL), left orbital middle frontal gyrus (ORBmid), right medial orbital superior frontal gyrus (ORBsupmed), right medial superior frontal gyrus (SFGmed), right Heschl gyrus (HES), left supramarginal gyrus (SMG), bilateral amygdala, right olfactory cortex (OLF), right parahippocampal gyrus (PHG), left middle temporal pole (TPOmid), and left superior occipital gyrus (SOG). Lower nodal degree was observed in the right orbital inferior frontal gyrus (ORBinf), right orbital middle frontal gyrus (ORBmid), left orbital superior frontal gyrus (ORBsup), left posterior cingulate gyrus (PCG), bilateral precuneus (PCUN), left postcentral gyrus (PoCG), right superior temporal pole (TPOsup), bilateral caudate nucleus (CAU), right median cingulate/paracingulate gyrus (DCG), left thalamus (THA), bilateral calcarine cortex (CAL), and bilateral lingual gyrus (LING). Detailed statistics and network assignments are presented in Table 2 and Figure 3A,B.

**TABLE 2:** Significant between‐group differences in nodal degree centrality.

Anatomical region	Whole region name	Network assignment	Effect Size (Cohen's d)	−log10(FDRp)
	CID > GSC			
IPL.R	Inferior parietal, but supramarginal and angular gyri	Attention	0.35	3.43
ORBmid.L	Middle frontal gyrus, orbital part	Attention	0.33	3.13
ORBsupmed.R	Superior frontal gyrus, medial orbital	Default mode	0.34	3.24
SFGmed.R	Superior frontal gyrus, medial	Default mode	0.45	5.04
HES.R	Heschl gyrus	Sensorimotor	0.35	3.27
SMG.L	Supramarginal gyrus	Sensorimotor	0.34	3.16
AMYG.L	Amygdala	Subcortical	0.44	4.88
AMYG.R	Amygdala	Subcortical	0.42	4.63
OLF.R	Olfactory cortex	Subcortical	0.42	4.63
PHG.R	Parahippocampal gyrus	Subcortical	0.39	3.99
TPOmid.L	Temporal pole: middle temporal gyrus	Subcortical	0.59	8.39
SOG.L	Superior occipital gyrus	Visual	0.50	5.95
	CID < GSC			
ORBinf.R	Inferior frontal gyrus, orbital part	Attention	0.40	4.09
ORBmid.R	Middle frontal gyrus, orbital part	Attention	0.32	3.03
ORBsup.L	Superior frontal gyrus, orbital part	Attention	0.33	3.09
PCG.L	Posterior cingulate gyrus	Default mode	0.31	2.85
PCUN.L	Precuneus	Default mode	0.36	3.55
PCUN.R	Precuneus	Default mode	0.33	3.13
PoCG.L	Postcentral gyrus	Sensorimotor	0.39	3.99
TPOsup.R	Temporal pole: superior temporal gyrus	Sensorimotor	0.33	3.10
CAU.L	Caudate nucleus	Subcortical	0.44	4.91
CAU.R	Caudate nucleus	Subcortical	0.61	8.77
DCG.R	Median cingulate and paracingulate gyri	Subcortical	0.34	3.13
THA.L	Thalamus	Subcortical	0.38	3.90
CAL.L	Calcarine fissure and surrounding cortex	Visual	0.33	3.09
CAL.R	Calcarine fissure and surrounding cortex	Visual	0.39	4.08
LING.L	Lingual gyrus	Visual	0.45	5.04
LING.R	Lingual gyrus	Visual	0.39	4.72

Nodal degree AUC values were compared between CID (n = 257) and GSC (n = 247) participants using two‐sample t tests across sparsity thresholds of 0.05–0.40 (step 0.05); results shown survived FDR correction (p_FDR < 0.05). Cohen's d is reported as the magnitude of the between‐group effect; effect direction is indicated separately by CID > GSC or CID < GSC. Network assignments are shown in the adjacent column. Abbreviations: IPL, inferior parietal lobule; ORBinf, inferior frontal gyrus, orbital part; ORBmid, middle frontal gyrus, orbital part; ORBsup, superior frontal gyrus, orbital part; ORBsupmed, superior frontal gyrus, medial orbital; SFGmed, superior frontal gyrus, medial; HES, Heschl gyrus; SMG, supramarginal gyrus; AMYG, amygdala; OLF, olfactory cortex; PHG, parahippocampal gyrus; TPOmid, middle temporal pole; TPOsup, superior temporal pole; SOG, superior occipital gyrus; PCG, posterior cingulate gyrus; PCUN, precuneus; PoCG, postcentral gyrus; CAU, caudate nucleus; DCG, median cingulate/paracingulate gyri; THA, thalamus; CAL, calcarine cortex; LING, lingual gyrus; L, left; R, right; CID, chronic insomnia disorder; GSC, good sleep controls; AUC, area under the curve; FDR, false discovery rate.

**FIGURE 3:**
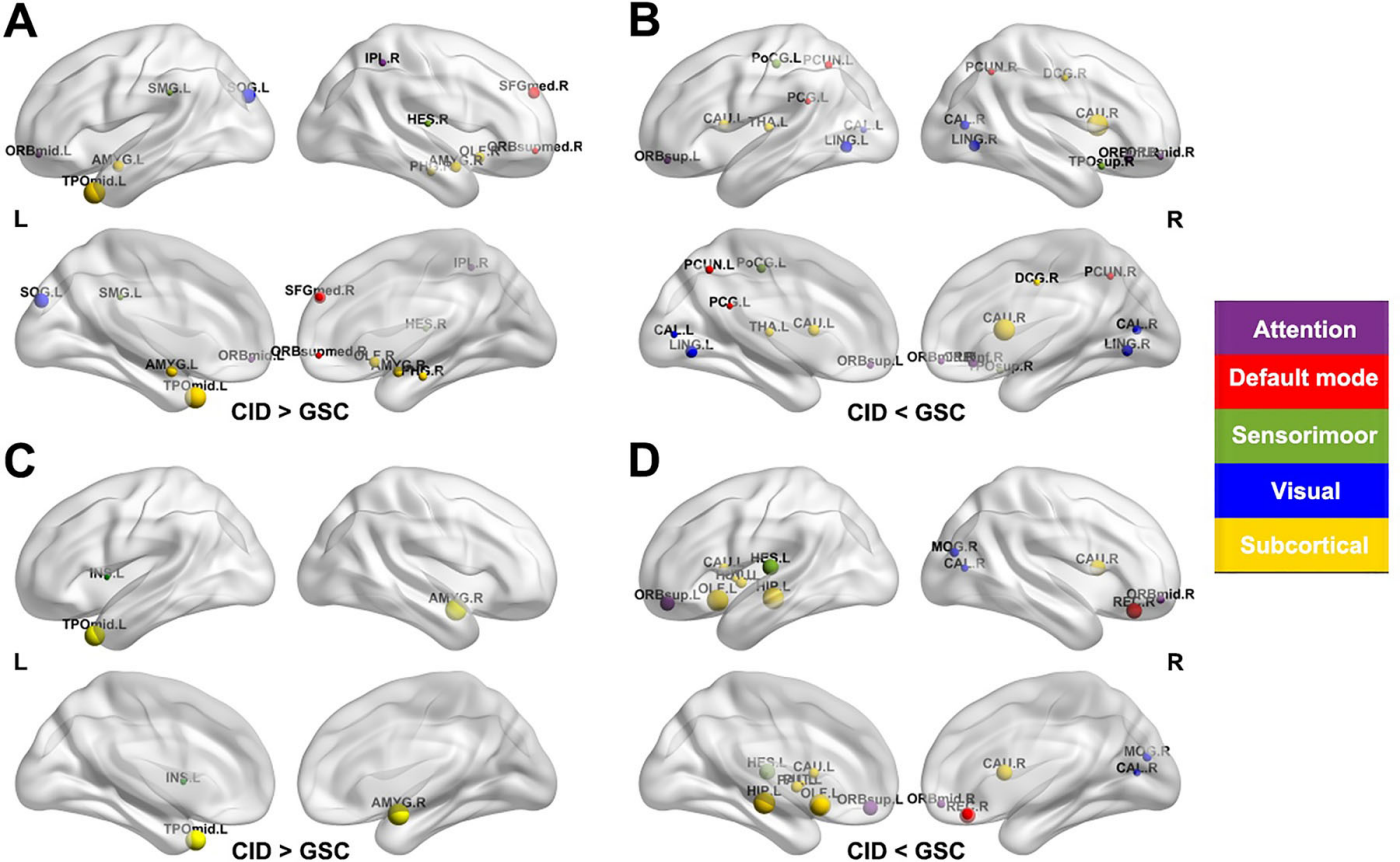
Group differences in nodal network topological properties. AUC values were calculated across sparsity thresholds of 0.05–0.40 (step 0.05) and compared between CID (n = 257) and GSC (n = 247) participants using two‐sample t tests with FDR correction (p_FDR < 0.05). (A) Regions with higher nodal degree in CID (CID > GSC). (B) Regions with lower nodal degree in CID (CID < GSC). (C) Regions with higher nodal betweenness in CID. (D) Regions with lower nodal betweenness in CID. Node size represents −log10(FDR‐corrected p), and node color denotes the network assignment shown in the legend (attention, default mode, sensorimotor, visual, or subcortical). Anatomical abbreviations are defined in Tables 2 and 3. CID, chronic insomnia disorder; GSC, good sleep controls; AUC, area under the curve; FDR, false discovery rate.

After FDR correction, higher nodal betweenness in the CID group was observed in the right amygdala, left insula, and left TPOmid, whereas lower nodal betweenness was observed in the right ORBmid, left ORBsup, right gyrus rectus (REC), left HES, bilateral CAU, left hippocampus (HIP), left OLF, left pallidum (PAL), left putamen (PUT), right CAL, and right middle occipital gyrus (MOG). Detailed statistics and network assignments are presented in Table 3 and Figure 3C,D. All 26 cerebellar AAL regions were included in the 116‐node network; none survived FDR correction in the nodal group comparisons.

**TABLE 3:** Significant between‐group differences in nodal betweenness centrality.

Anatomical region		Whole region name	Network assignment	Effect Size (Cohen's d)	−log10(FDRp)
	CID > GSC				
AMYG.R		Amygdala	Subcortical	0.54	7.67
INS.L		Insula	Sensorimotor	0.37	3.74
TPOmid.L		Temporal pole: middle temporal gyrus	Subcortical	0.53	7.37
CID < GSC					
ORBmid.R		Middle frontal gyrus, orbital part	Attention	0.54	4.28
ORBsup.L		Superior frontal gyrus, orbital part	Attention	0.40	7.67
REC.R		Gyrus rectus	Default mode	0.57	8.19
HES.L		Heschl gyrus	Sensorimotor	0.33	3.06
CAU.L		Caudate nucleus	Subcortical	0.45	5.23
CAU.R		Caudate nucleus	Subcortical	0.58	8.37
HIP.L		Hippocampus	Subcortical	0.68	11.40
OLF.L		Olfactory cortex	Subcortical	0.68	11.40
PAL.L		Lenticular nucleus, pallidum	Subcortical	0.58	8.45
PUT.L		Lenticular nucleus, putamen	Subcortical	0.40	5.84
CAL.R		Calcarine fissure and surrounding cortex	Visual	0.37	3.74
MOG.R		Middle occipital gyrus	Visual	0.42	4.67

Nodal betweenness AUC values were compared between CID (n = 257) and GSC (n = 247) participants using two‐sample t tests across sparsity thresholds of 0.05–0.40 (step 0.05); results shown survived FDR correction (p_FDR < 0.05). Cohen's d is reported as the magnitude of the between‐group effect; effect direction is indicated separately by CID > GSC or CID < GSC. Network assignments are shown in the adjacent column. Abbreviations: AMYG, amygdala; INS, insula; TPOmid, middle temporal pole; ORBmid, middle frontal gyrus, orbital part; ORBsup, superior frontal gyrus, orbital part; REC, gyrus rectus; HES, Heschl gyrus; CAU, caudate nucleus; HIP, hippocampus; OLF, olfactory cortex; PAL, pallidum; PUT, putamen; CAL, calcarine cortex; MOG, middle occipital gyrus; L, left; R, right; CID, chronic insomnia disorder; GSC, good sleep controls; AUC, area under the curve; FDR, false discovery rate.

### 3.4 Clinical Associations of Altered Network Metrics in the CID Group

After FDR correction, higher individual nodal degree AUC values in the right TPOsup were associated with higher PSQI scores (r = 0.22, *p* = 2.47 × 10^−^^4^) within the CID group. Lower nodal degree values in the bilateral caudate (left: r = −0.19, *p* = 1.77 × 10^−^^3^; right: r = −0.27, *p* = 8.09 × 10^−^^6^) and left putamen (r = −0.37, *p* = 4.15 × 10^−^^7^) were associated with higher SDS scores, whereas higher nodal degree in the right ORBinf (r = 0.29, *p* = 1.85 × 10^−^^6^) and right amygdala (r = 0.21, *p* = 4.79 × 10^−^^4^), and higher nodal betweenness in the left insula (r = 0.21, *p* = 6.69 × 10^−^^4^), were associated with higher SDS scores (Figure 4). For anxiety symptoms, lower nodal degree in the bilateral thalamus (left: r = −0.45, *p* = 5.99 × 10^−^^1^ ^5^; right: r = −0.45, *p* = 1.70 × 10^−^^14^), lower nodal betweenness in the bilateral caudate (left: r = −0.26, *p* = 2.30 × 10^−^^5^; right: r = −0.21, *p* = 6.90 × 10^−^^4^) and left putamen (r = −0.27, *p* = 9.63 × 10^−^^6^), and higher nodal degree in the bilateral ORBinf (left: r = 0.27, *p* = 6.20 × 10^−^^6^; right: r = 0.29, *p* = 8.90 × 10^−^^7^) and higher nodal betweenness in the left insula (r = 0.24, *p* = 1.40 × 10^−^^4^) were associated with SAS scores (Figure 5). No other FDR‐significant associations were observed, including associations with illness duration.

**FIGURE 4:**
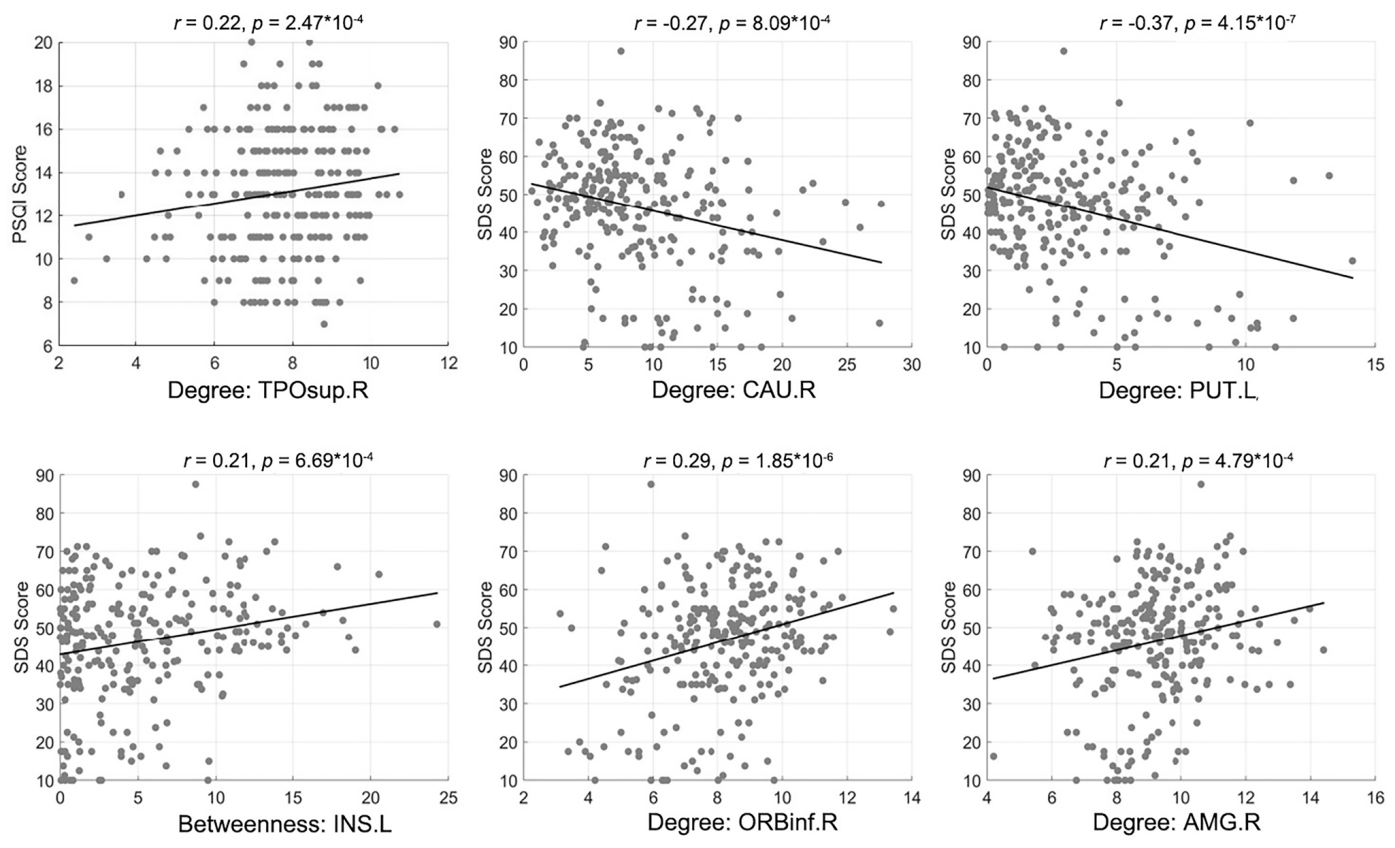
Associations of individual nodal metrics with PSQI and SDS scores in the CID group (n = 257). Scatterplots show representative FDR‐significant partial correlations between subject‐level nodal AUC metrics and clinical scores after adjustment for age, sex, and education. Higher right TPOsup degree was associated with higher PSQI (r = 0.22, *p* = 2.47 × 10^−^^4^). For SDS, right caudate degree (r = −0.27, *p* = 8.09 × 10^−^^6^) and left putamen degree (r = −0.37, *p* = 4.15 × 10^−^^7^) were negatively associated, whereas left insula betweenness (r = 0.21, *p* = 6.69 × 10^−^^4^), right ORBinf degree (r = 0.29, *p* = 1.85 × 10^−^^6^), and right amygdala degree (r = 0.21, *p* = 4.79 × 10^−^^4^) were positively associated. All displayed associations survived FDR correction. PSQI, Pittsburgh Sleep Quality Index; SDS, Zung Self‐Rating Depression Scale; TPOsup, superior temporal pole; CAU, caudate nucleus; PUT, putamen; INS, insula; ORBinf, inferior frontal gyrus, orbital part; AMYG, amygdala; L, left; R, right; AUC, area under the curve; FDR, false discovery rate.

**FIGURE 5:**
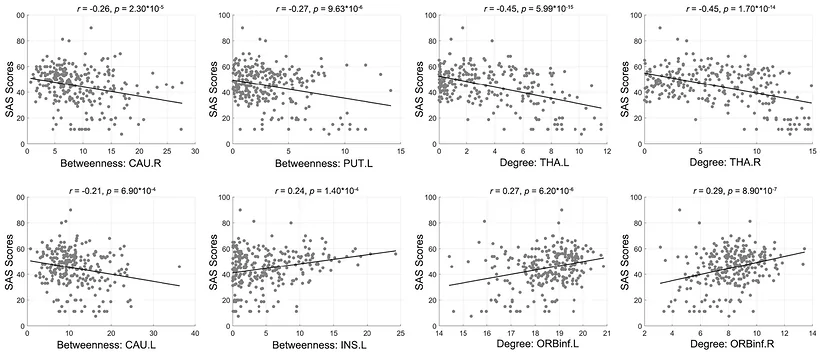
Associations of individual nodal metrics with SAS scores in the CID group (n = 257). Scatterplots show FDR‐significant partial correlations between subject‐level nodal AUC metrics and SAS after adjustment for age, sex, and education. Lower bilateral thalamic degree (left: r = −0.45, *p* = 5.99 × 10^−^^1^ ^5^; right: r = −0.45, *p* = 1.70 × 10^−^^14^), lower bilateral caudate betweenness (left: r = −0.26, *p* = 2.30 × 10^−^^5^; right: r = −0.21, *p* = 6.90 × 10^−^^4^), and lower left putamen betweenness (r = −0.27, *p* = 9.63 × 10^−^^6^) were associated with higher SAS; higher bilateral ORBinf degree (left: r = 0.27, *p* = 6.20 × 10^−^^6^; right: r = 0.29, *p* = 8.90 × 10^−^^7^) and higher left insula betweenness (r = 0.24, *p* = 1.40 × 10^−^^4^) were also associated with higher SAS. All displayed associations survived FDR correction. SAS, Zung Self‐Rating Anxiety Scale; CAU, caudate nucleus; PUT, putamen; THA, thalamus; INS, insula; ORBinf, inferior frontal gyrus, orbital part; L, left; R, right; AUC, area under the curve; FDR, false discovery rate.

Exploratory mediation analyses confirmed that PSQI severity was significantly associated with both SAS and SDS scores; however, none of the candidate nodal degree or betweenness metrics showed a significant indirect effect in the PSQI → network metric → SAS or PSQI → network metric → SDS models. All bootstrap 95% confidence intervals for the indirect effects included zero, and no mediation effect survived FDR correction (all *q* > 0.05). Thus, the present cross‐sectional data did not provide evidence that the identified structural network metrics statistically mediated the association between insomnia severity and affective symptom burden.

## 4 Discussion

In this study, we combined DTW‐based individualized structural covariance mapping with graph theory to characterize iSCN alterations in a large CID sample and to examine their associations with depressive and anxiety symptoms. Compared with GSC participants, CID patients showed reduced global and local efficiency and reduced small‐world‐related properties. At the nodal level, CID was characterized by a mixed pattern of higher and lower centrality across regions involved in emotion regulation, arousal, sensory processing, and subcortical integration. Importantly, several subject‐level nodal metrics were associated with SDS and SAS symptom severity, supporting a link between atypical structural network organization and affective symptom burden in CID.

The observed reductions in global efficiency (Eg) and local efficiency (Eloc), together with diminished small‐world properties (Sigma and Gamma), indicate disruption of the architectural organization of structural networks in CID. Small‐world topology reflects an economical balance between local specialization and global integration (Bassett and Bullmore 2017), which appears to be compromised in CID (Z. Li et al. 2018). This altered organization may reflect the cumulative neurobiological effects of chronic sleep disturbance on large‐scale structural coordination and is broadly consistent with previous reports of altered functional network topology after chronic sleep restriction (Farahani et al. 2019). Such an inefficient network configuration may constrain flexible coordination among cognitive, emotional, and sensory systems, potentially contributing to daytime dysfunction and emotional dysregulation in CID.

Importantly, total gray matter volume, total white matter volume, and TIV did not differ significantly between groups. This suggests that the observed iSCN abnormalities were not simply attributable to a generalized reduction in whole‐brain tissue volume, but instead reflected differences in the distributed pattern of interregional morphological coordination.

At the nodal level, CID showed both increased and decreased centrality across regions implicated in emotion, arousal, sensory processing, and self‐referential functions. These directional changes should not be interpreted as uniformly beneficial or detrimental. Structural covariance reflects coordinated variation in morphology that may arise from shared developmental, trophic, experiential, or disease‐related influences; therefore, increased centrality may reflect altered or maladaptive coupling as well as possible compensatory reorganization, whereas decreased centrality may indicate reduced structural coordination. Because the present data are cross‐sectional, we cannot determine whether any specific increase or decrease is compensatory, maladaptive, or causal. The amygdala and orbitofrontal findings, together with reduced centrality in the caudate, thalamus, and posterior cingulate/precuneus, are therefore interpreted as evidence of atypical structural organization involving emotion/arousal, sensory‐gating, and default‐mode‐related systems rather than as direct evidence of improved or impaired regional function (Gent, Bassetti & Adamantidis, 2018; Killgore et al. 2023).

The positive association between right superior temporal pole (TPOsup) nodal degree and PSQI score further implicates sensory‐processing systems in the subjective severity of sleep disturbance in CID. The temporal pole contributes to multimodal integration and self‐referential and socioemotional processing (Olson et al. 2007). A greater nodal degree in this region may reflect atypical structural coordination related to heightened sensory salience or hyperarousal, potentially contributing to difficulty disengaging from environmental or internal stimuli around sleep. This interpretation is compatible with the hyperarousal model of insomnia (Gong et al. 2023; Riemann et al. 2010; Baglioni et al. 2014), but should remain cautious because structural covariance does not directly index neural activity or causal communication.

Notably, depressive and anxiety symptoms showed distinct yet overlapping associations with subcortical and limbic network topology. The caudate, putamen, and thalamus were inversely associated with affective symptom severity, whereas orbitofrontal, amygdala, and insular metrics showed positive associations. These patterns are consistent with involvement of corticostriatal‐thalamic, reward, salience, and interoceptive systems in affective symptoms accompanying insomnia (Gong et al. 2021; Peters et al. 2016; Spellman and Liston 2020; Šimić et al. 2021; Zou et al. 2021). The comparatively strong association between left putamen degree and SDS may reflect the putamen's position within striatal circuits supporting reward, motivation, and action selection, processes frequently disturbed in depressive symptomatology (Bishop et al. 2009; Du et al. 2020); however, this region‐specific association should be regarded as hypothesis‐generating rather than evidence of a unique causal role. In addition, no network metric was significantly associated with illness duration after multiple‐comparison correction. This may suggest that the observed topology is more closely related to current symptom burden than to simple illness chronicity, but the cross‐sectional design, retrospective estimation of illness duration, and clinical heterogeneity preclude interpreting these measures as definitive trait markers. The exploratory mediation analyses did not identify significant indirect effects of these network metrics on the relationship between PSQI and either SAS or SDS. Accordingly, the present findings support an association between iSCN topology and affective symptoms but do not establish a mediating mechanism linking insomnia severity to anxiety or depressive symptom burden.

The DTW‐based iSCN framework provides an individualized alternative to conventional group‐level structural covariance analysis (Gratton et al. 2020). By allowing elastic alignment of regional GMV vectors with different lengths, DTW yields a subject‐specific interregional similarity estimate while avoiding the requirement for group‐level covariance calculation (Sun et al. 2024). We analyzed graph metrics across sparsity thresholds from 0.05 to 0.40, a range used in prior individual morphological network studies, and summarized each metric by its AUC. This threshold‐integrated approach reduces sensitivity to any single sparsity choice, although it does not eliminate all threshold‐related uncertainty. The large sample and FDR correction further improve statistical precision, but independent replication remains necessary.

Several limitations should be acknowledged. First, the cross‐sectional design precludes causal inference regarding whether iSCN alterations predispose to CID, result from chronic sleep disturbance, or reflect correlated affective symptoms. The exploratory mediation analyses were likewise statistical rather than causal and yielded no significant indirect effects. Second, major depressive disorder and generalized anxiety disorder were excluded by clinical diagnostic interview and neurologist consensus, and participants with standardized SDS or SAS scores ≥ 70 were additionally excluded. Nevertheless, SDS and SAS are dimensional self‐rating scales rather than diagnostic instruments; the elevated scores observed in CID should therefore be interpreted as subthreshold affective symptom burden, and residual unrecognized psychiatric comorbidity cannot be completely excluded. Third, years of education differed significantly between groups. Although education was included as a covariate in the brain‐clinical correlation and mediation analyses, the primary between‐group network comparisons were based on two‐sample t tests and were not adjusted for education. Because education may influence cognitive reserve and brain morphology, residual confounding cannot be excluded, and the group‐level findings require confirmation in better‐matched cohorts or covariate‐adjusted sensitivity analyses. Fourth, participants with overt brain abnormalities on conventional MRI were excluded, but white matter hyperintensity burden was not quantitatively modeled; subtle cerebrovascular changes may therefore contribute to interindividual morphological variability. Fifth, although all 26 cerebellar AAL nodes were included, no cerebellar region survived FDR correction in the present nodal comparisons; this absence should not be interpreted as evidence that the cerebellum is uninvolved in CID. Finally, the analysis focused on gray matter covariance and did not integrate white matter connectivity, neurochemical measures, or functional networks. Longitudinal, multimodal, and independently replicated studies will be required to determine the biological meaning and temporal stability of these iSCN alterations.

## 5 Conclusion

In conclusion, CID is associated with altered individualized structural covariance network topology at both global and nodal levels. Atypical nodal organization in cortical‐subcortical systems involved in emotion, arousal, sensory processing, and reward is associated with depressive and anxiety symptom severity. These findings identify structural network correlates of affective symptom burden in CID, while the negative exploratory mediation results and cross‐sectional design emphasize that directionality and mediation cannot be inferred. Longitudinal and multimodal studies are needed to establish specificity, temporal ordering, and clinical utility.

## Author Contributions

**Liang Gong**: conceptualization, investigation, funding acquisition, Writing – original draft, Writing – review and editing, supervision. **Duan Liu**: data curation, investigation, visualization, methodology, writing – review and editing, writing – original draft. **Bei Zhang**: data curation, writing – review and editing. **Haoyu Li**: data curation, writing – review and editing. **Yuan He**: data curation, writing – review and editing. **Ronghua Xu**: funding acquisition, writing – review and editing, resources. **Wenjun Zhou**: project administration, conceptualization, validation, writing – review and editing. **Chunhua Xi**: project administration, validation, conceptualization, funding acquisition, writing – review and editing.

## Funding

This work was supported by National Natural Science Foundation of China (No. 82371479, 82001803), Sichuan Provincial Science and Technology Department Project in China (2024ZYD0136, 2026YFHZ0202), Chengdu Medical College Graduate Education Teaching Reform Project (YJG202507), Anhui Provincial Department of Education Research Project (2025AHGXZK30517).

## Data Availability

The analysis code and associated datasets for this study are available upon reasonable request.
